# In silico screening and analysis of nonsynonymous SNPs in human *CYP1A2* to assess possible associations with pathogenicity and cancer susceptibility

**DOI:** 10.1038/s41598-021-83696-x

**Published:** 2021-03-02

**Authors:** Leila Navapour, Navid Mogharrab

**Affiliations:** Biophysics and Computational Biology Laboratory (BCBL), Department of Biology, College of Sciences, Shiraz University, Shiraz, Iran

**Keywords:** Computational biology and bioinformatics, Cancer, Cancer, Molecular biology, Structural biology, Genetics

## Abstract

Cytochrome P450 1A2 (CYP1A2) is one of the main hepatic CYPs involved in metabolism of carcinogens and clinically used drugs. Nonsynonymous single nucleotide polymorphisms (nsSNPs) of this enzyme could affect cancer susceptibility and drug efficiency. Hence, identification of human *CYP1A2* pathogenic nsSNPs could be of great importance in personalized medicine and pharmacogenetics. Here, 176 nsSNPs of human *CYP1A2* were evaluated using a variety of computational tools, of which 18 nsSNPs were found to be associated with pathogenicity. Further analysis suggested possible association of 9 nsSNPs (G73R, G73W, R108Q, R108W, E168K, E346K, R431W, F432S and R456H) with the risk of hepatocellular carcinoma. Molecular dynamics simulations revealed higher overall flexibility, decreased intramolecular hydrogen bonds and lower content of regular secondary structures for both cancer driver variants G73W and F432S when compared to the wild-type structure. In case of F432S, loss of the conserved hydrogen bond between Arg137 and heme propionate oxygen may affect heme stability and the observed significant rise in fluctuation of the CD loop could modify CYP1A2 interactions with its redox partners. Together, these findings propose *CYP1A2* as a possible candidate for hepatocellular carcinoma and provide structural insights into how cancer driver nsSNPs could affect protein structure, heme stability and interaction network.

## Introduction

The genome of two individuals, except for identical twins, shares 99.9% identity and only differs by 0.1%. Although, this value seems very low, it is responsible for about 3 million differences among 3.2 billion base pairs^1^. The most abundant genetic variations in the human genome are single nucleotide polymorphisms (SNPs) which play a significant role in the phenotypic diversity, interindividual differences in susceptibility to complex diseases and drug reactions^1,2^. However, a small number of the SNPs is associated with pathogenicity that must be distinguished from a pool of neutral variants. Although experimental techniques provide the most accurate and reliable approaches for assessing the consequences of a substitution, analysis of all SNPs in human genome or even in a single gene is a major challenge for researchers due to the complex, time-consuming and costly experimental procedures^3^. Therefore, in silico computational approaches have attracted considerable interest of biologists, as they make it possible to screen a large number of SNPs in a relatively short time and low cost, and to prioritize them for further experimental and clinical tests. Moreover, the structure–function relationship studies by molecular dynamics (MD) simulations could elucidate the molecular mechanisms of diseases and may provide valuable insights into the diagnosis as well as treatment^4–7^.

The human cytochrome P450 (CYP) superfamily enzymes are the most important enzymes of the phase I xenobiotic metabolism and known as one of the highly polymorphic proteins^8,9^. Single nucleotide polymorphisms in the enzymes of this superfamily play an important role in differences between individuals in response to drugs and other xenobiotics as well as the susceptibility to develop various diseases^10^. Among 18 cytochrome P450 families encoded by the human genome, members of CYP1 family are particularly important due to their major contribution to the metabolism of carcinogenic compounds such as polycyclic aromatic hydrocarbons (PAHs)^11–13^. This family of CYPs has three members CYP1A1, CYP1A2 and CYP1B1 grouped into A and B subfamilies^11,13^.

The human *CYP1A2* gene is located on the long arm of chromosome 15 (15q24.1) that spans seven exons. The CYP1A2 protein is exclusively expressed in liver and plays an important role in metabolism of heterocyclic and aromatic amines, caffeine and melatonin^14–18^. This enzyme is also responsible for hepatic metabolism of many clinically used drugs such as tacrine, zolpidem, clozapine, theophylline and so on^19–23^, while the other two enzymes of this family do not have significant role in drug metabolism.

The human *CYP1A2* gene encodes a heme-binding protein composed of 516 residues. The three-dimensional (3D) structure of the protein covering residues 34–513 has been determined in complex with the inhibitor α-naphthoflavone, while the structure of the N-terminal transmembrane helical domain is lost in this crystal structure^14^. According to the crystal structure (PDB ID: 2HI4^14^), CYP1A2 holds fifteen α-helices and five β-sheets^14^. The iron atom of the heme prosthetic group is coordinated by the Cys458 of the protein moiety which belongs to the consensus signature of cytochrome P450 proteins (PROSITE signature PS00086)^24^. In addition, arginine 137 (R137) from C helix is hydrogen bonded to the heme propionate oxygen and further stabilizes its position.

CYPalleles is a web page which was developed to standardize the nomenclature of human cytochrome P450 alleles (http://www.cypalleles.ki.se/^9^). It also provides genetic information and the molecular effect of the variants on the enzyme activity. More than 20 alleles have been reported for *CYP1A2* gene in CYPalleles, among them, CYP1A2*6 (R431W), CYP1A2*8 (R456H), CYP1A2*11 (F186L), CYP1A2*15 (P42R) and CYP1A2*16 (R377Q) are the most studied alleles^25–29^. Nevertheless, structural or functional consequences of the vast majority of nsSNPs for *CYP1A2* recorded by the NCBI dbSNP database have not yet been determined. Since CYP1A2 is one of the main hepatic CYPs involved in the bioactivation of carcinogens and metabolism of clinically used drugs, SNPs of this enzyme could affect cancer susceptibility or drug efficiency. Therefore, the identification and evaluation of *CYP1A2* pathogenic nonsynonymous SNPs (nsSNPs) are of major importance. This is also helpful in personalized medicine and optimization of drug treatment to achieve the most efficiency and least side effects. In this study, nsSNPs of *CYP1A2* gene were evaluated by computational tools to identify pathogenic nsSNPs. We also performed MD simulation to assess how these nsSNPs affect the protein structure.

## Methods

### Data collection

The human CYP1A2 protein sequence was obtained from UniProt database^30^ (UniProt ID: P05177). SNP data for *CYP1A2* gene were retrieved from NCBI dbSNP^31^ build 150. All nucleotide positions were related to GRCh37.p13 (hg19) annotation release 105. The three-dimensional structure of the CYP1A2 protein (PDB ID: 2HI4^14^) was downloaded from Protein Data Bank (https://www.rcsb.org^32^).

### In silico evaluation of nsSNPs

In silico evaluation of *CYP1A2* nsSNPs was performed using a variety of computational tools in a stepwise fashion where the output of each step was served as the input for the next one. SIFT^33^, PROVEAN^34^, MutationAssessor^35^, EFIN^36^, LRT^37^, FATHMM-MKL^38^, PhD-SNP^39^, and CADD^40^ are sequence-based predictors which could be easily applied to amino acid or nucleotide sequences. PolyPhen2^41^, SNAP2^42^, SuSPect^43^, PMUT^44^ and MutPred2^45^ are sequence- and structure-based tools which utilize the user-provided sequence information and the self-extracted structural features to predict if SNPs are associated with functional effects.

We categorized the tools into three groups (Table 1). SIFT^33^, PROVEAN^34^, MutationAssessor^35^, EFIN^36^, LRT^37^, FATHMM-MKL^38^, CADD^40^, PolyPhen2^41^ and SNAP2^42^ were used to predict the impact of the nsSNPs on the protein function. PhD-SNP^39^, SuSPect^43^, PMUT^44^, MutPred2^45^ and VEST-4^46^ were employed to assess the likelihood that a variant is pathogenic. CHASM-3.1^47^ was used to identify possible cancer driver variants. All prediction scores were received directly from their own web servers except for VEST-4 and CHASM-3.1 which were fetched from CRAVAT^48^ server. In addition to the score, VEST-4 and CHASM-3.1 also assign a *p*-value to each variation and an approximate false discovery rate (FDR) for each *p*-value. The *p*-value denotes the probability that benign/passenger variant is misclassified as a pathogenic/driver.

**Table 1:** Classification of the methods used for in silico evaluation of *CYP1A2* gene nsSNPs.

Method	Prediction category	Prediction result
SIFT	Functional impact	Deleterious/Tolerated
PolyPhen2	Functional impact	Probably damaging/Possibly damaging/Benign
PROVEAN	Functional impact	Deleterious/Neutral
MutationAssessor	Functional impact	High/Medium/Low/Neutral
SNAP2	Functional impact	Effect/Neutral
LRT	Functional impact	Deleterious/Neutral
EFIN	Functional impact	Damaging/Neutral
FATHMM-MKL	Functional impact	Deleterious/Neutral
CADD	Functional impact	Deleterious/Neutral
PhD-SNP	Deleteriousness	Disease/Neutral
SuSPect	Deleteriousness	Disease-causing/Neutral
MutPred2	Pathogenicity	Pathogenic/Benign
PMUT	Pathogenicity	Disease/Neutral
VEST-4	Pathogenicity	Pathogenic/Benign
CHASM-3.1	Cancer susceptibility	Driver/Passenger

### Evolutionary conservation analysis

The evolutionary conservation of amino acid positions was calculated with ConSurf^49,50^ web server which assigns a score between 1 (most variable position) and 9 (most conserved position) to each amino acid position. The protein sequence similarity searching was performed against UNIREF-90 in which CSI-BLAST (Context-Specific Iterated-Basic Local Alignment Search Tool), 3 and 0.0001 were set for homolog search algorithm, number of iteration and E-value cutoff, respectively.

### Prediction of transmembrane helix

The TMHMM 2.0 (Transmembrane Hidden Markov Model)^51^ web server was used to predict transmembrane helices. The TMHMM incorporates hydrophobicity, charge bias, helix lengths and grammatical constraints into prediction of various regions of a transmembrane protein.

### Molecular dynamics simulation

All MD simulations were conducted by GROMACS package version 5.0.5^52^ using the CHARMM36 force field^53^. The crystal structure of the CYP1A2 protein (PDB ID: 2HI4^14^) was used as the starting structure for wild-type (WT) protein after removing the ligand (alpha-naphthoflavone) atomic coordinates. The initial structure of the variant proteins was generated from WT structure using mutate tool of Swiss-Pdb Viewer v4.1.0^54^. The proteins were immersed in a cubic box of TIP3P water molecules. An adequate number of water molecules was replaced by counter ions to neutralize the systems. Each neutralized system was then subjected to steepest descent energy minimization until the maximum force fell below 500 kJ mol^−1^ nm^−1^. In order to equilibrate the solvent and ions around the proteins, two position-restrained MD simulations were carried out. The temperature and pressure of the systems were controlled at 300 K and 1 bar by V-rescale thermostat^55^ and Parrinello-Rahman barostat^56^, respectively. After equilibration, each system was subjected to 200 ns (ns) unrestrained MD simulation considering the similar conditions as two previous position-restraint simulations. The LINCS algorithm^57^ was used to constrain the bonds with hydrogen atoms and the particle mesh Ewald method^58^ was employed for long range electrostatic interactions. The Cut-off distance for the Lennard–Jones, short-range and long-range electrostatic interaction was set to 12 Å. A time step of 2 fs was used for integrating Newton's equations of motion.

### Trajectory analysis and visualization

Most of the trajectory analyses reported in this study were performed by built-in utilities of GROMACS package version 5.0.5^52^. The root mean square deviation (RMSD), root mean square fluctuation (RMSF), radius of gyration (Rg), and intramolecular hydrogen bonds were analyzed using gmx rms, gmx rmsf, gmx gyrate and gmx hbond of GROMACS package, respectively. The secondary structure content of the proteins was calculated as a function of time using the DSSP program^59^. The principal component analysis (PCA) was conducted using gmx covar and gmx anaeig. To perform free energy landscape (FEL) analysis, all-atom RMSD with respect to the average structure and radius of gyration were initially obtained for the analyzed time frames and then were employed by gmx sham module of GROMACS for calculation of Gibbs free energy as well as construction of FEL. A conformation with minimum free energy was extracted as the representative structure for visualization. The three-dimensional structures of the proteins were visualized using Chimera 1.11^60^. The CaPTURE program^61^ was used to explore cation-pi interactions of the snapshots extracted from the MD trajectories.

## Results

### The SNP dataset

**Figure 1:**
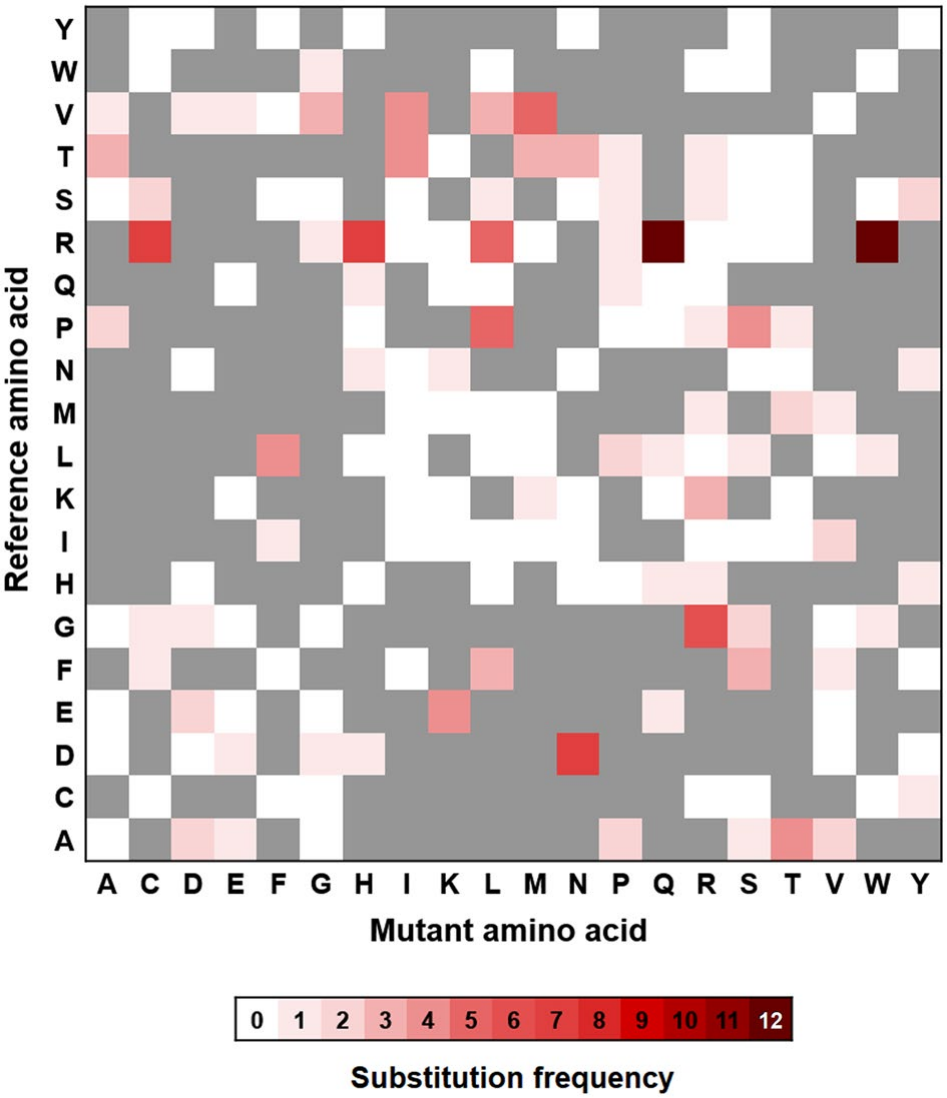
The Amino acid substitution heatmap. The one-letter codes of amino acids at the left and bottom side of the map correspond to the reference and mutant amino acids, respectively. A color index (white to red) was assigned for amino acid substitutions according to the number of their occurrences ranged from 0 to 12. The gray blocks show the amino acid replacements that are not allowed to occur by single nucleotide substitution.

The nsSNPs of human *CYP1A2* gene were retrieved from the NCBI dbSNP database^31^ build 150. The nsSNPs which met at least one of the following criteria in the validation method were entered to the evaluation: (1) sequenced in 1000Genome project (1000G), (2) validated by multiple independent submissions to the refSNP cluster, (3) validated by frequency or genotype data, (4) genotyped by HapMap project, (5) validated by submitter confirmation, and (6) observed in at least two chromosome apiece. The nsSNPs which have no information on validation method (did not have any of the mentioned criteria) were excluded. Among them, there were four known alleles of *CYP1A2* which were listed in CYPalleles including P42R (CYP1A2*15), S212C (CYP1A2*12), R377Q (CYP1A2*16) and N397H (CYP1A2*18). Since these alleles have been frequently studied, we made an exception for these nsSNPs and included them in our analyses. Totally, 176 nsSNPs were prepared for analysis (Supplementary Table S1). More than half of the nsSNPs occured in exon 2 (n = 94) and the others were mapped in exons 3 (n = 10), 4 (n = 8), 5 (n = 17), 6 (n = 14) and 7 (n = 33). The G to A transition is the most frequent nucleotide substitution (29.5%) found among all analyzed variations followed by C to T (23.9%), A to G (6.8%) and C to A (6.8%). At the protein level, the most common amino acids as the reference and missense were Arg (n = 45) and Leu (n = 17), respectively. The replacements of Arg with Trp (n = 12, 6.8%), Gln (n = 12, 6.8%), Cys (n = 7, 4.0%), and His (n = 7, 4.0%) and substitution of Asp by Asn (n = 7, 4.0%) are the most frequent amino acid substitutions (Fig. 1).

### In silico evaluation of nsSNPs

**Figure 2:**
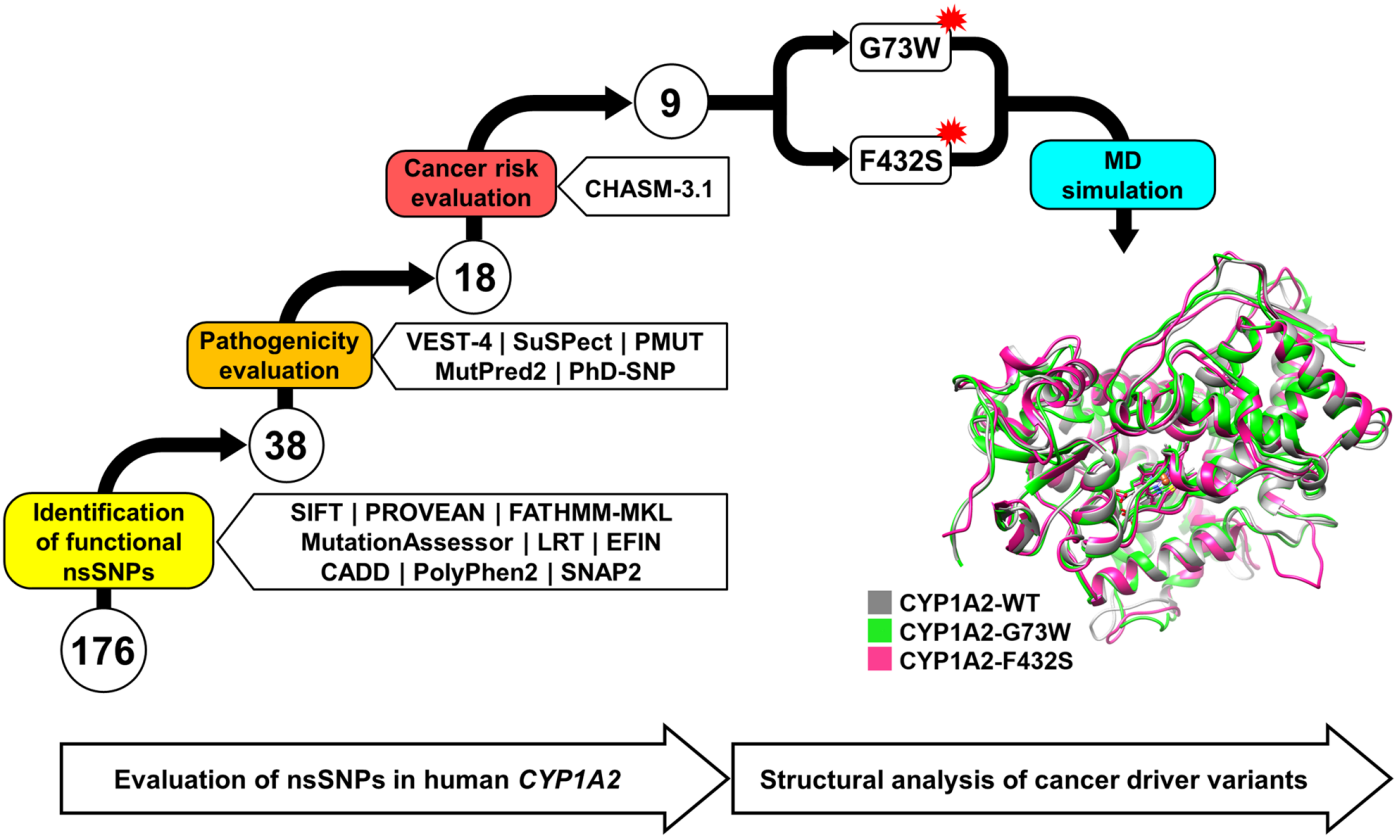
**S**chematic representation of the stepwise evaluation of *CYP1A2* gene nonsynonymous single nucleotide polymorphisms (nsSNPs). A total of 176 nsSNPs were entered into the analysis by a variety of computational tools. In first step, 38 out of 176 nsSNPs were found to be associated with functional effects. Among them, 18 nsSNPs were predicted as pathogenic. Finally, 9 nsSNPs (G73R, G73W, R108Q, R108W, E168K, E346K, R431W, F432S and R456H) were also found to be cancer drivers. G73W and F432S cancer driver variants were then subjected to 200 ns molecular dynamics simulation. Conformations with minimum free energy were extracted as the representative structure for wild-type, G73W and F432S CYP1A2 proteins using FEL analysis and were visualized and superimposed using UCSF Chimera 1.11 (www.cgl.ucsf.edu/chimera).

**Figure 3:**
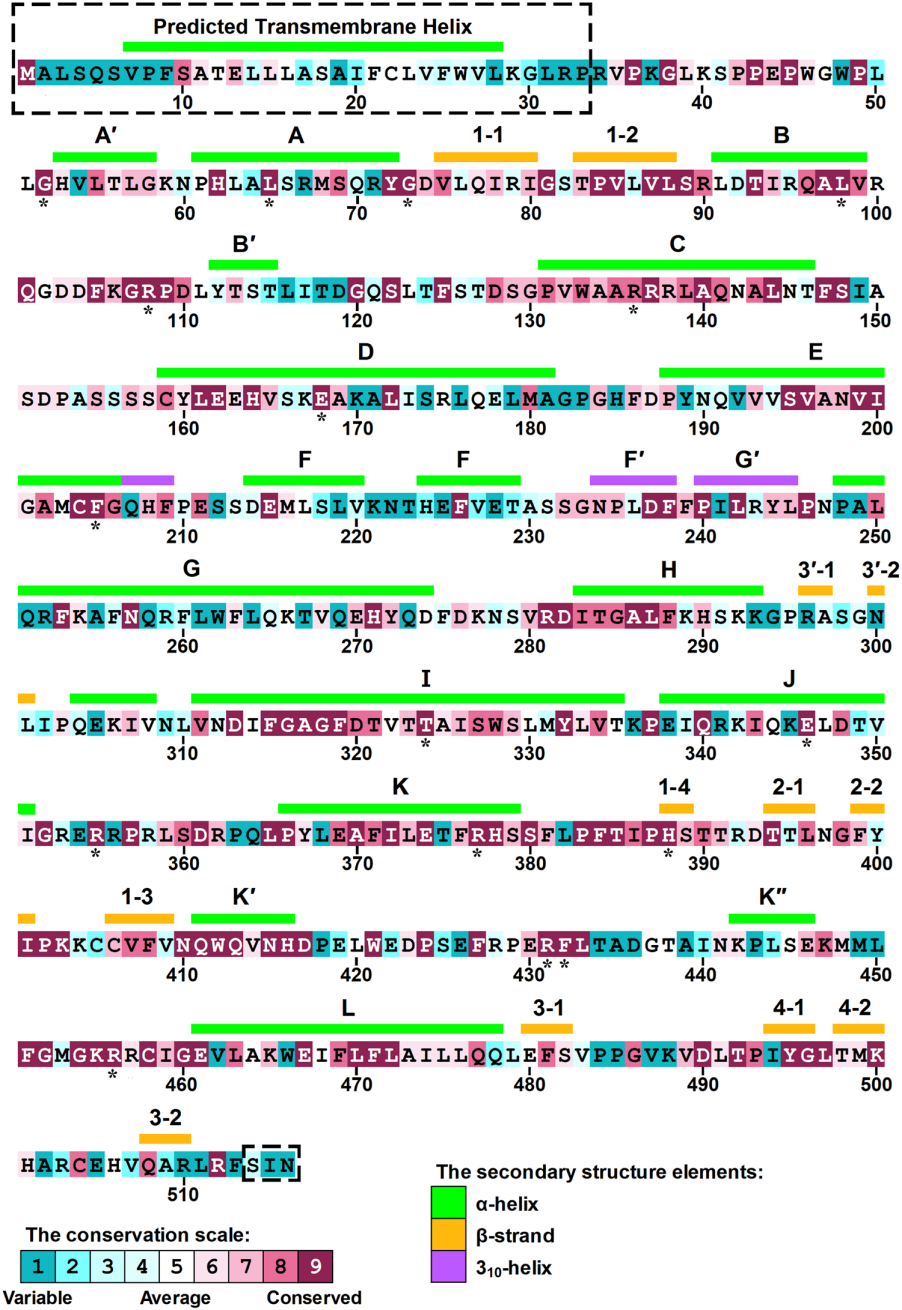
The conservation profile of CYP1A2 amino acid positions calculated by ConSurf (consurf.tau.ac.il). Each amino acid position is scored based on the conservation score obtained from multiple sequence alignment. The higher the score, the more conserved is the position. The uppercase letters (helices) and numbers (strands) represent the regular secondary structure elements. The structure of the areas enclosed by dashed lines has not yet been determined. The positions labeled by black stars indicate amino acid positions of the 18 nsSNPs which predicted as pathogenic.

As shown in Fig. 2, a total of 176 nsSNPs for human *CYP1A2* gene were evaluated in a multi-step framework. A variant must be voted by all of the tools to proceed to the next step of the analysis. Firstly, all nsSNPs were evaluated by SIFT, PROVEAN, MutationAssessor, LRT, FATHMM-MKL, EFIN, CADD, PolyPhen2 and SNAP2 to identify functional nsSNPs. As a result, 38 nsSNPs were agreed to be associated with functional effects by all of the used methods (Supplementary Table S2). Subsequently, the isolated nsSNPs were subjected to pathogenicity evaluation using SuSPect, MutPred2, PMUT, PhD-SNP and VEST-4. 18 out of the 38 examined nsSNPs including G52R, L65P, G73R, G73W, L98Q, R108Q, R108W, R136C, E168K, F205V, T324R, E346K, R355W, R377Q, H388Y, R431W, F432S and R456H were classified as pathogenic by all five methods (Table 2). The evolutionary conservation profile was calculated for the amino acid position of these pathogenic variants using ConSurf^49,50^ web server. The conservation scores calculated by this server range from 1 to 9, and discriminate between highly variable and highly conserved positions, respectively. The results included one position (136) with score of 8 and fifteen positions (52, 65, 73, 98, 108, 168, 205, 324, 346, 355, 377, 388, 431, 432 and 456) with score of 9 (Fig. 3), indicating that almost all of the pathogenic nsSNPs affect evolutionary conserved positions in CYP1A2 protein.

**Table 2:** Pathogenicity evaluation of functional CYP1A2 nsSNPs.

Variant	PhD-SNP	SuSPect	MutPred2	PMUT	VEST-4
	Pred (S)	Pred (S)	Pred (S)	Pred (S)	Pred (S, *P*-value, FDR)
P36S	N (0.23)	N (16)	N (0.38)	N (0.36)	N (0.48, 0.194, 0.55)
P42R	P (0.53)	P (96)	P (0.69)	P (0.91)	N (0.48, 0.196, 0.55)
**G52R**	**P (0.85)**	**P (95)**	**P (0.95)**	**P (0.91)**	**P (0.97, 0.003, 0.10)**
P61L	P (0.55)	N (42)	P (0.58)	N (0.48)	N (0.69, 0.077, 0.35)
**L65P**	**P (0.86)**	**P (92)**	**P (0.90)**	**P (0.86)**	**P (0.93, 0.006, 0.10)**
**G73R**	**P (0.86)**	**P (88)**	**P (0.91)**	**P (0.91)**	**P (0.97, 0.003, 0.10)**
**G73W**	**P (0.90)**	**P (94)**	**P (0.92)**	**P (0.91)**	**P (0.96, 0.003, 0.10)**
R79C	P (0.80)	P (78)	N (0.33)	N (0.48)	N (0.17, 0.582, 0.90)
V85M	N (0.37)	P (53)	P (0.59)	N (0.24)	N (0.46, 0.207, 0.55)
**L98Q**	**P (0.84)**	**P (93)**	**P (0.91)**	**P (0.91)**	**P (0.96, 0.003, 0.10)**
**R108Q**	**P (0.83)**	**P (86)**	**P (0.82)**	**P (0.91)**	**P (0.98, 0.002, 0.10)**
**R108W**	**P (0.89)**	**P (97)**	**P (0.90)**	**P (0.91)**	**P (0.98, 0.002, 0.10)**
F125L	N (0.42)	N (41)	P (0.73)	N (0.30)	P (0.91, 0.008, 0.10)
**R136C**	**P (0.69)**	**P (54)**	**P (0.71)**	**P (0.63)**	**P (0.76, 0.046, 0.25)**
R137Q	P (0.82)	P (92)	P (0.75)	P (0.87)	N (0.71, 0.069, 0.35)
R138C	P (0.91)	P (75)	P (0.62)	P (0.78)	N (0.35, 0.291, 0.60)
V165G	P (0.73)	P (64)	P (0.65)	P (0.68)	N (0.53, 0.172, 0.55)
**E168K**	**P (0.86)**	**P (75)**	**P (0.71)**	**P (0.57)**	**P (0.77, 0.045, 0.25)**
**F205V**	**P (0.87)**	**P (78)**	**P (0.77)**	**P (0.63)**	**P (0.93, 0.006, 0.10)**
F238S	P (0.61)	N (47)	P (0.83)	N (0.28)	P (0.95, 0.004, 0.10)
R243C	P (0.74)	P (59)	N (0.40)	N (0.31)	N (0.40, 0.244, 0.55)
T324I	P (0.86)	P (54)	P (0.71)	N (0.40)	P (0.95, 0.004, 0.10)
**T324R**	**P (0.92)**	**P (85)**	**P (0.83)**	**P (0.78)**	**P (0.97, 0.003, 0.10)**
**E346K**	**P (0.87)**	**P (93)**	**P (0.72)**	**P (0.90)**	**P (0.96, 0.003, 0.10)**
R355Q	P (0.57)	N (44)	P (0.70)	N (0.41)	N (0.47, 0.203, 0.55)
**R355W**	**P (0.82)**	**P (71)**	**P (0.77)**	**P (0.74)**	**P (0.81, 0.029, 0.20)**
**R377Q**	**P (0.79)**	**P (95)**	**P (0.77)**	**P (0.90)**	**P (0.94, 0.005, 0.10)**
I386F	N (0.40)	P (57)	P (0.72)	N (0.30)	P (0.93, 0.006, 0.10)
**H388Y**	**P (0.64)**	**P (78)**	**P (0.85)**	**P (0.85)**	**P (0.98, 0.002, 0.10)**
**R431W**	**P (0.82)**	**P (96)**	**P (0.81)**	**P (0.90)**	**P (0.89, 0.012, 0.10)**
**F432S**	**P (0.87)**	**P (95)**	**P (0.91)**	**P (0.90)**	**P (0.97, 0.003, 0.10)**
K447M	N (0.47)	P (56)	P (0.64)	P (0.70)	N (0.57, 0.146, 0.55)
**R456H**	**P (0.86)**	**P (79)**	**P (0.78)**	**P (0.89)**	**P (0.94, 0.005, 0.10)**
R457P	P (0.87)	P (62)	P (0.92)	P (0.87)	N (0.70, 0.074, 0.35)
R457W	P (0.79)	P (80)	P (0.79)	P (0.87)	N (0.54, 0.164, 0.55)
E461K	P (0.91)	N (28)	P (0.80)	P (0.58)	N (0.73, 0.059, 0.30)
A473D	P (0.89)	P (84)	P (0.59)	P (0.91)	N (0.72, 0.064, 0.30)
T498N	P (0.73)	P (64)	P (0.51)	P (0.51)	N (0.48, 0.195, 0.55)

Pred: Prediction, S: Score, *P*-value: Probability that a benign variant is misclassified as pathogen, FDR: False discovery rate, P: Pathogenic, N: Neutral/Benign.

The nsSNPs classified as pathogenic by all five methods are highlighted in bold.

The filtered pathogenic variants were further analyzed with CHASM-3.1^47^ to assess possible association with cancer susceptibility. CHASM-3.1 consists of cancer-specific classifiers which allow to predict cancer driver variants depending on a particular cancer type. Since CYP1A2 is a hepatic enzyme, we selected liver-viral (hepatocellular carcinoma) to compute the cancer driver scores. The results reported in Table 3 revealed a possible association with hepatocellular carcinoma for G73R, G73W, R108Q, R108W, E168K, E346K, R431W, F432S and R456H variants (*P*-value < 0.05).

**Table 3:** Assessing the cancer susceptibility of pathogenic CYP1A2 nsSNPs using CHASM-3.1.

Variant	Prediction	Score	*P*-value	FDR
G52R	Passenger	0.50	0.080	0.70
L65P	Passenger	0.49	0.100	0.70
G73R	Cancer driver	0.63	0.013	0.30
G73W	Cancer driver	0.62	0.014	0.30
L98Q	Passenger	0.48	0.110	0.75
R108Q	Cancer driver	0.67	0.006	0.20
R108W	Cancer driver	0.68	0.005	0.20
R136C	Passenger	0.42	0.190	0.80
E168K	Cancer driver	0.60	0.021	0.35
F205V	Passenger	0.43	0.178	0.80
T324R	Passenger	0.36	0.316	0.80
E346K	Cancer driver	0.63	0.012	0.30
R355W	Passenger	0.53	0.056	0.60
R377Q	Passenger	0.52	0.061	0.60
H388Y	Passenger	0.51	0.071	0.65
R431W	Cancer driver	0.65	0.007	0.20
F432S	Cancer driver	0.66	0.007	0.20
R456H	Cancer driver	0.72	0.002	0.20

*P*-value: Probability that a passenger variant is misclassified as driver, FDR: False discovery rate.

### Evaluation of nsSNPs occurring in transmembrane helix

The CYP1A2 is a membrane-bound protein which is anchored to the endoplasmic reticulum membrane through an N-terminal transmembrane helix. However, to date, no complete structure for CYP1A2 including this region has been determined. Hence, the sequence of CYP1A2 protein was submitted to TMHMM server v2.0 to predict the transmembrane helix. According to the server’s estimation, the transmembrane helix includes residues 7 to 28. Nine substitutions including S10L, L15F, S18C, S18Y, A19P, F21L, F25C, F25S and V27M have occurred in the transmembrane helical region, none of which were found to be associated with pathogenicity. Moreover, evolutionary conservation analysis of the nsSNPs located in this transmembrane helix did not found any conserved amino acid position other than Ser10 (Fig. 3).

### Molecular dynamics simulation

**Figure 4:**
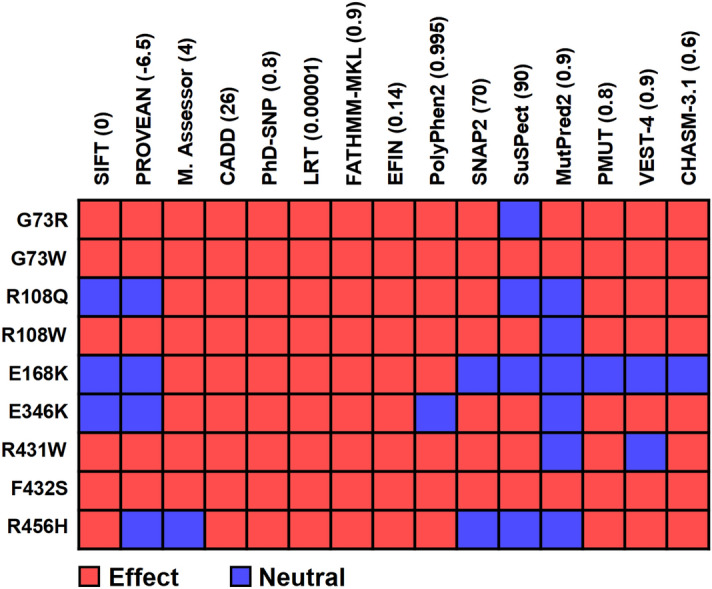
Evaluation of the cancer driver nsSNPs by all methods with modified thresholds. The numbers in parentheses refer to the user-defined thresholds.

In order to determine which of the cancer driver nsSNPs should be subjected to MD simulation, we used all evaluation tools with stringent threshold of effectiveness/deleteriousness (Fig. 4). As a result, two cancer driver nsSNPs G73W and F432S voted by all the tools were selected for the structural evaluation by MD simulation. The structure of CYP1A2 (PDB ID: 2HI4^14^) after removal of the ligand (alpha-naphthoflavone) was used as the wild-type (WT) protein. The initial structure of the G73W and F432S variants was obtained by substitution of the corresponding residues in the WT structure. Finally, variant and WT structures were subjected to 200 ns MD simulation to explore possible impacts of the substitutions on protein structure.

**Figure 5:**
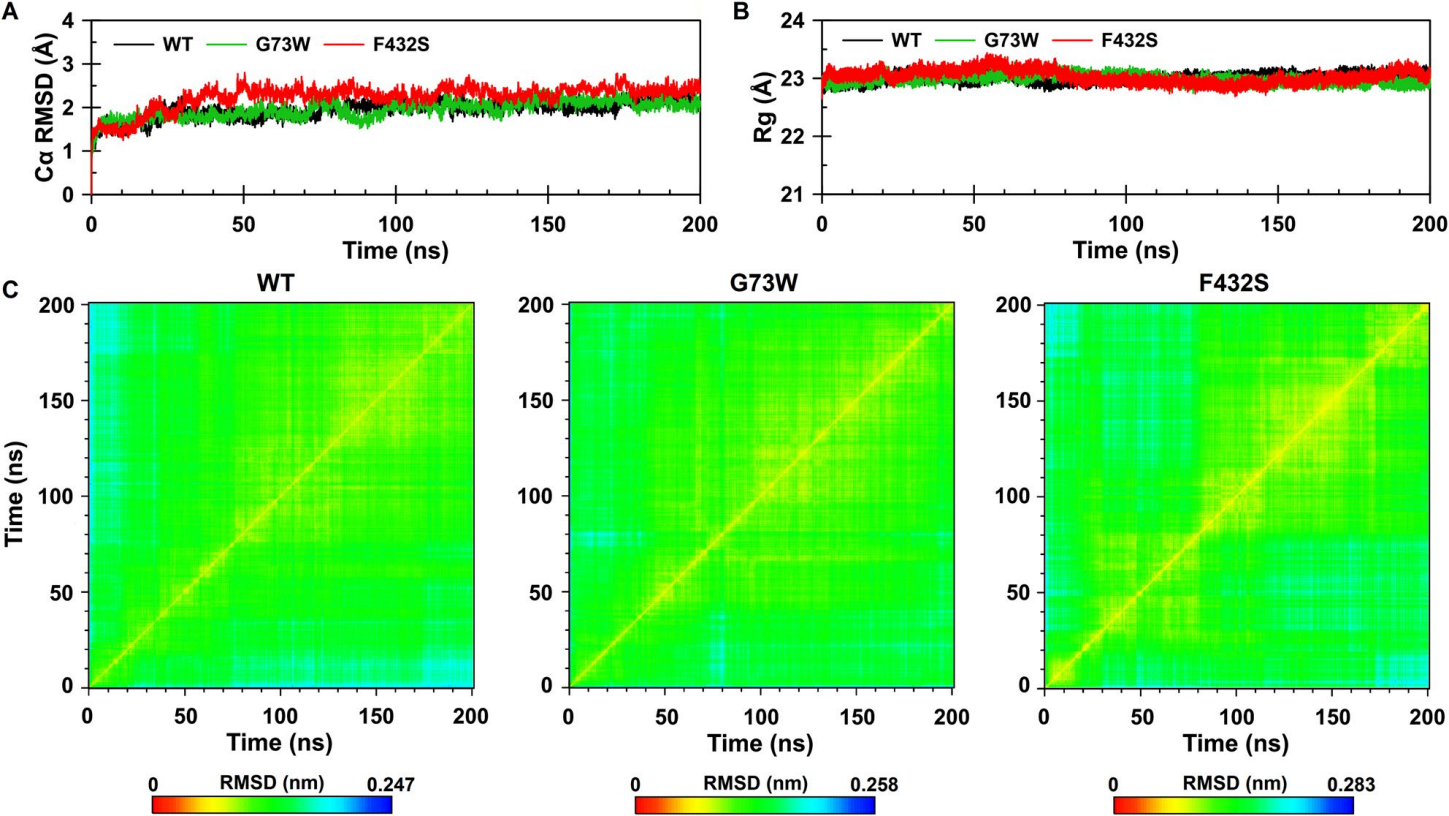
**(A)** Time dependence of the root mean square deviation and **(B)** Radius of gyration calculated for Cα atoms. WT, G73W and F432S are shown in black, green and red, respectively. **(C)** Two-dimensional root mean square deviation (2D-RMSD) plots calculated for Cα atoms as a function of the simulation time. The plots are color-coded according to RMSD values (nm).

Root mean square deviation (RMSD) of the alpha carbon (Cα) atoms for each frame with respect to the starting (1D-RMSD) and to all other frames (2D-RMSD) as well as radius of gyration (Rg) along the simulation time were calculated (Fig. 5). By comparing the 1D-RMSD trend it was found that the G73W (1.95 ± 0.20 Å) behaves more or less similar to the WT (1.94 ± 0.21 Å), whereas the F432S demonstrates minor deviation in the Cα atom positions (2.24 ± 0.28 Å). The 2D-RMSD plots indicate that WT and G73W variant converged to relatively stable conformations after about 40 ns of simulations (Fig. 5C), while for F432S variant, such a stable conformation was achieved after about 80 ns, suggesting that F432S has experienced more structural changes before running out into a stable structure (Fig. 5C). The measurement of Rg as a function of the simulation time also implied that the structures converged after about 80 ns (Fig. 5B). Taking these findings together and to be statistically comparable, the analyses were focused on those trajectories obtained from the last 120 ns of simulations (from 80 to 200 ns) for all the three proteins.

**Figure 6:**
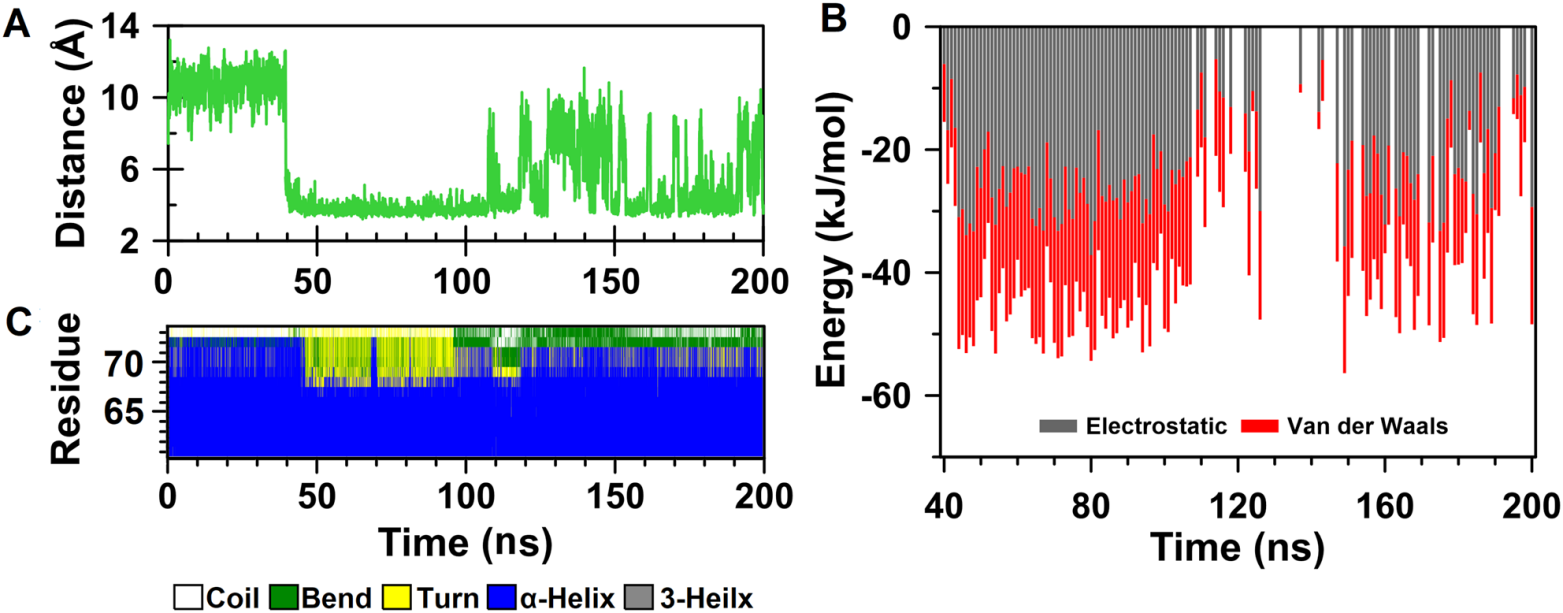
Local structural changes resulting from G73W substitution. **(A)** Time evaluation of the distance between aromatic moiety of Trp73 and guanidinium group of Arg90. **(B)** Binding energies of W73-R90 cation-pi interaction calculated with CaPTURE program. The total binding energy is equal to the electrostatic plus the van der Waals interaction energies (E_total_ = E_elec_ + E_vdW_). **(C)** Time-dependent secondary structure of the A helix. The occurrence of secondary structure elements is indicated by using a color code.

In order to gain more insight into the local structural changes around substitution sites, we extracted a conformation with minimum free energy as the representative structure using free energy landscape analysis from the last 120 ns of each MD simulation. Gly73 is located in a short loop just after A helix (residues 61–72). Substitution of this residue by tryptophan renders the indole ring of Trp to be captured by the positive charge of the guanidinium group of Arg90. As a result, a cation-pi interaction formed between Trp73 and Arg90 after about 39 ns of the simulation. The distance between the indole ring and guanidinium group is maintained at about 4 Å for 70 ns, after that the magnitude of the fluctuations increased (Fig. 6A). In this regard, 161 snapshots were extracted at every 1 ns from 40 to 200 ns of the simulation time and explored for cation-pi interactions by CaPTURE program^61^. The results taken from CaPTURE also confirmed the formation of cation-pi interaction between Trp73 and Arg90, although it was attenuated after 120 ns (Fig. 6B). On the other hand, analysis of the secondary structure showed the C-terminal end of the A helix became unstable after establishment of cation-pi interaction between Trp73 and Arg90 (Fig. 6C). The next substitution site, Phe432, is located in a 3_10_ helix flanked by helices K' and K". In WT structure, most of the residues located within a radius of 5 Å from side chain of Phe432 are nonpolar residues, of which, Ala370, Leu373, Trp421, Ile440, Leu444 and Met448 have been shown to be involved in van der Waals interactions with the aromatic moiety of Phe432. By comparing WT and F432S representative structures, it was cleared that substitution of serine residue with a small polar side chain for F432 has led to disappearance of these interactions.

**Figure 7:**
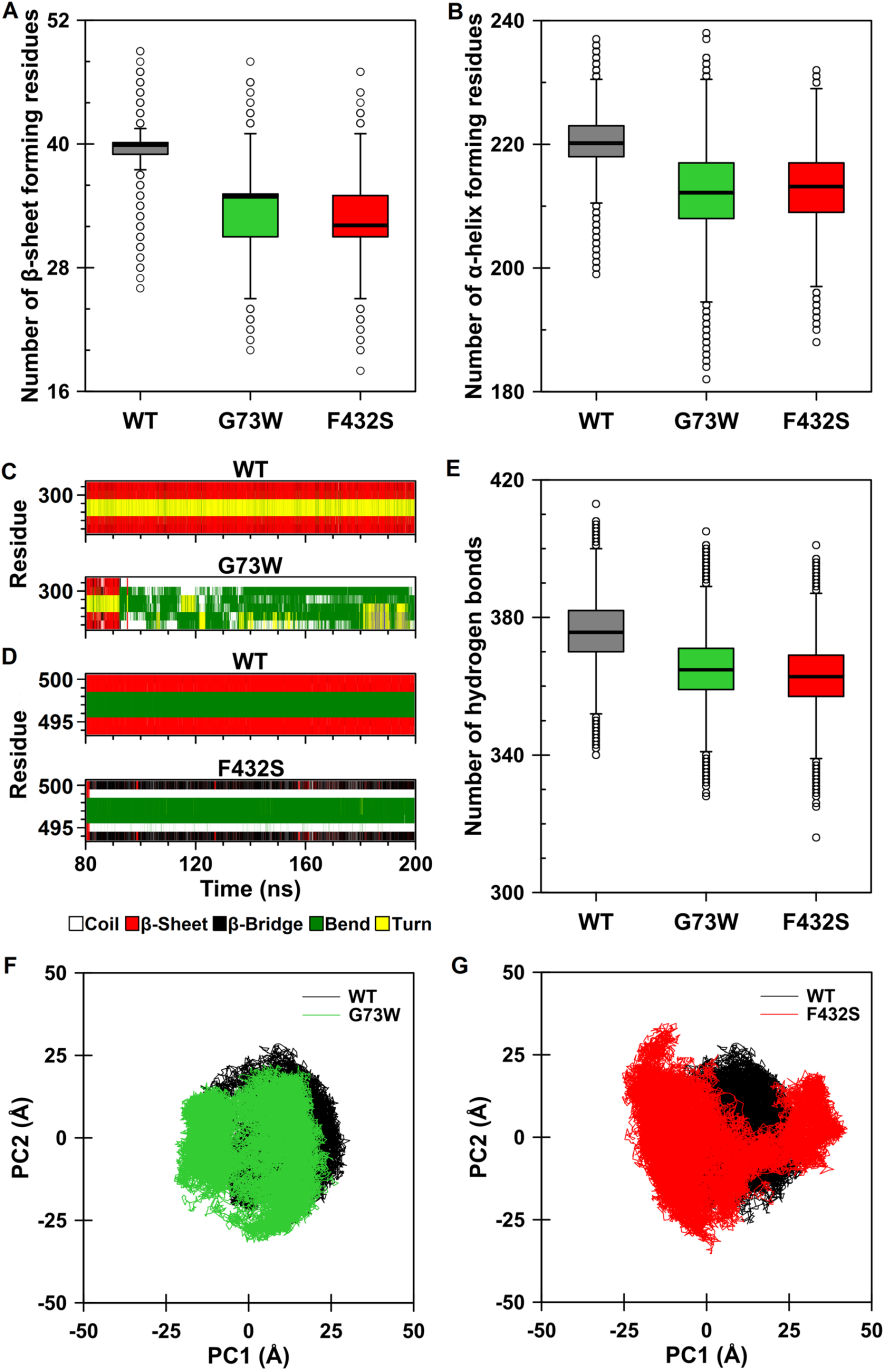
Trajectory analysis of the wild-type, G73W and F432S CYP1A2 proteins during the analyzed time frames (last 120 ns). **(A,B)** Number of β-sheet and α-helix forming residues. Box plots for WT, G73W and F432S are shown in dark gray, green and red, respectively. **(C,D)** Time-dependent secondary structure profile of β-sheets 3′ and 4 for G73W and F432S variants, respectively. **(E)** Comparison of the intramolecular hydrogen bonds. **(F,G)** Projection of the motion of the protein in phase space along the first two principal eigenvectors. Comparison of G73W (green) and F432S (red) with WT (black) are presented.

We also conducted further analyses to explore the overall structural changes upon substitutions. The secondary structure content of the proteins was also measured during the analyzed time window. Both variants showed a decrease in the β-sheet (Fig. 7A) and α-helical content (Fig. 7B). The average number of β-sheet forming residues was reduced from 40 ± 2 in WT to 34 ± 3 in G73W variant and 33 ± 3 in F432S variant. The average number of residues participating in α-helix was also decreased from 220 ± 4 in WT to 212 ± 7 and 213 ± 6 in G73W and F432S, respectively. Detailed analysis of secondary structure elements revealed disruption of β-sheet 3′ in G73W variant (Fig. 7C) and β-sheet 4 in F432S variant (Fig. 7D). The results implied that no α-helix structure was completely lost, they were just shortened by one or more residues.

On the other hand, analysis of hydrogen bonds implied a decrease in the number of intramolecular hydrogen bonds in both variants as the average number of hydrogen bonds was reduced from 376 ± 9 in WT to 365 ± 9 and 363 ± 9 in G73W and F432S variants, respectively (Fig. 7E). It was also observed that the number of hydrogen bonds with occupancy above 70% has decreased from 264 in WT to 237 and 249 in G73W and F432S variants, respectively. The reduction in the number and strength of hydrogen bonds suggested a gain in the overall flexibility of the variants upon substitutions. So, to examine whether these substitutions affect protein overall flexibility, we performed principal component analysis (PCA). The Eigenvectors and eigenvalues were obtained from diagonalization of the covariance matrices of the Cα atoms, and the principal components were generated by projecting the trajectories on the respective eigenvectors (Fig. 7F and 7G). The trace of the diagonalized covariance matrix was found to be 530.27 Å^2^, 693.11 Å^2^ and 931.91 Å^2^ for WT, G73W and F432S variants, respectively, confirming an increase in the overall flexibility of the variants, of which the increase in the F432S variant is more drastic compared to that of G73W.

**Figure 8:**
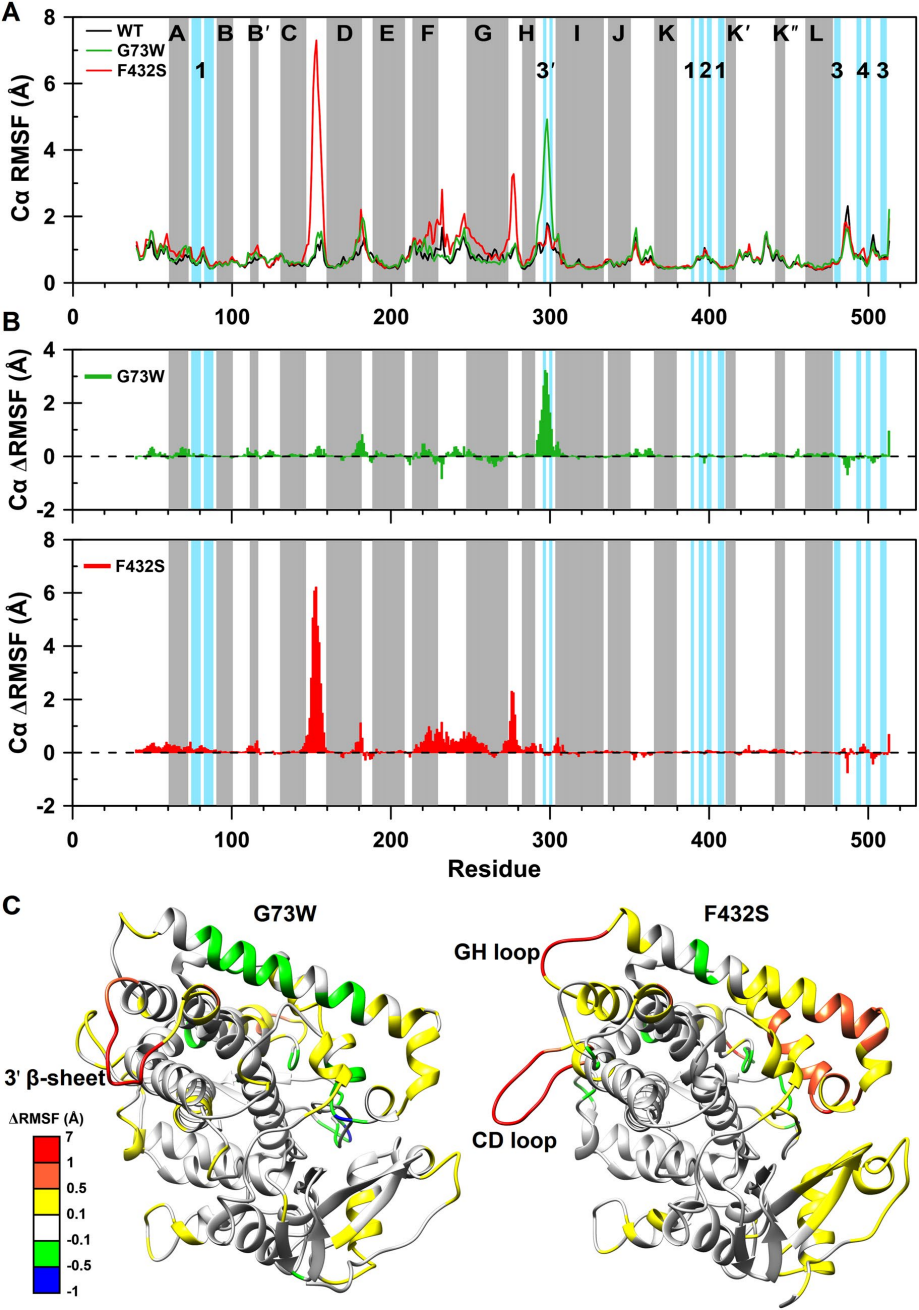
**(A)** Root mean square fluctuation (RMSF) of the Cα atoms as a function of residue number. The gray and blue blocks indicate α-helices (denoted with letters) and β-strands (denoted with numbers), respectively. WT protein is shown in black, G73W in green and F432S in red. **(B)** Change in RMSF (ΔRMSF) of the Cα atoms for G73W (green) and F432S (red) with respect to the WT as a function of residue number. Positive and negative values of ΔRMSF indicate more and less flexible residues compared to the WT ones, respectively. **(C)** Three-dimensional representation of G73W and F432S structures colored based on ΔRMSF values. A Conformation with minimum free energy was extracted as the representative structure for G73W and F432S using FEL analysis and were visualized using UCSF Chimera 1.11 (www.cgl.ucsf.edu/chimera). The position of β-sheet 3′ in G73W, CD and GH loops in F432S, showing significant differences in ΔRMSF values are marked on corresponding structures.

In order to provide more insight into the protein structural flexibility, RMSF of the Cα atoms as a function of residue number was calculated over the last 120 ns (Fig. 8A). The RMSF graph has been highlighted with color blocks indicating α-helices and β-strands according to CYP1A2 crystallographic structure. The differences in per-residue RMSF (ΔRMSF) for G73W and F432S Cα atoms with respect to the WT were also measured and visualized in Fig. 8B. Positive values indicate more flexible residues and negative values show less flexible residues compared to those of WT. As seen in Figs. 8B and 8C, a significant increase in flexibility was measured for β-sheet 3′ and its flanking loops of G73W variant. Disruption of β-sheet 3′ due to breaking of two hydrogen bonds between Ala297 and Asn300 confirmed the higher flexibility in this region (Fig. 7C). In case of F432S variant, a sharp increase in fluctuation of the CD loop was particularly significant (Figs. 8B and 8C). Increased flexibility was also observed in F helix, FG loop, G helix and GH loop.

**Figure 9:**
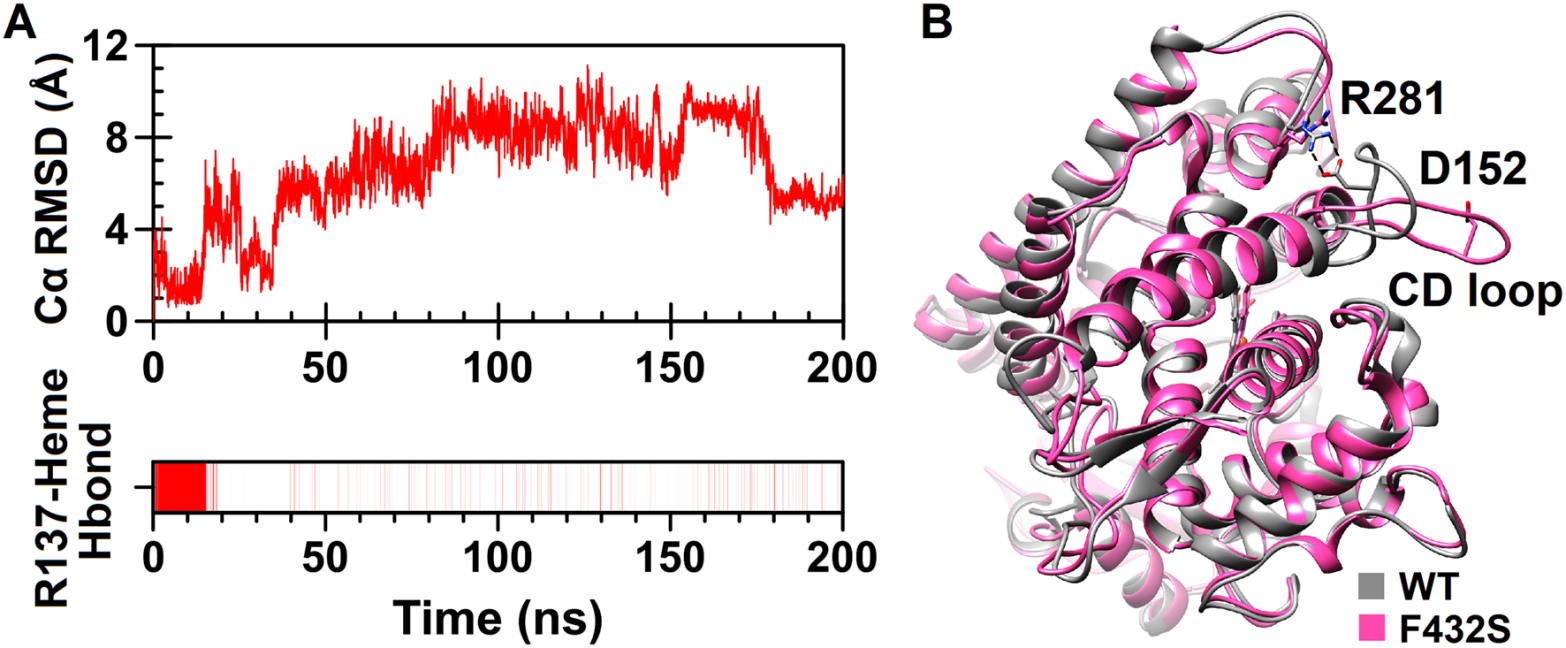
Structural changes caused by F432S substitution. **(A)** Evaluation of the Cα RMSD as a function of time for the CD loop (upper plot) and existence map for the hydrogen bond between Arg137 of the C helix and heme propionate (lower plot) are shown. Presence of the R137-heme hydrogen bond is shown by vertical red lines. **(B)** The positions of the CD loop and D152-R281 salt bridge in superimposed structures of the wild-type CYP1A2 (gray) and F432S (pink). UCSF Chimera 1.11 (www.cgl.ucsf.edu/chimera) was used for superposition and three-dimensional visualization of the structures.

Calculation of RMSD for Cα of the CD loop during the entire course of the F432S simulation demonstrated displacement of this loop after about 14 ns of the simulation (Fig. 9A). In addition, monitoring of the CD loop interactions revealed that F432S has lost the hydrogen bonding network in this region of the protein (Supplementary Table S3). The salt bridge between Asp152 from this loop and Arg281 from GH loop has also disrupted (Fig. 9B). The removal of these interactions thought to be the reason for displacement and higher mobility of the CD loop as well as GH loop in F432S variant. Another notable change was the significant weakening of the conserved hydrogen bond between Arg137 of the C helix and the heme propionate oxygen which occurred shortly after CD loop movement (Fig. 9A). By looking at the results described for CD loop, it may be concluded that displacement of the CD loop together with its increased flexibility have induced breakage of the Arg137-heme hydrogen bond.

## Discussion

In this study, we performed a comprehensive in silico evaluation to identify *CYP1A2* gene pathogenic nsSNPs using a wide variety of computational tools. To our knowledge only one study has been carried out to evaluate the nsSNPs of human *CYP1A2* gene. Wang et al. using two tools SIFT and PolyPhen analyzed the functional impact of thirty-three nsSNPs of *CYP1A2* gene and reported eleven nsSNPs as damaging substitutions^62^. We expanded our study to include more nsSNPs and hypothesized that a more reliable and accurate estimate of a substitution consequence could be provided by using a variety of computational methods that follow different approaches to distinguish between pathogenic and neutral variants. Although all predictive methods have been developed to estimate whether a given substitution has functional/pathogenic effect, it does not necessarily mean that they can elucidate the mechanism how the SNPs affect protein function or cause disease. This question could be explored using other experimental or computational techniques including MD simulation.

To test our hypothesis, we initially annotated the nsSNPs using a variety of computational methods to distinguish between functional and neutral variants. Assessing the pathogenicity of functional nsSNPs identified 18 pathogenic nsSNPs. Evolutionary conservation analysis indicated that almost all of the pathogenic nsSNPs occupy conserved amino acid positions. Moreover, the results obtained from CHASM-3.1 revealed a possible association between G73R, G73W, R108Q, R108W, E168K, E346K, R431W, F432S and R456H with risk of developing hepatocellular carcinoma.

The results of this study are in fairly good agreement with those published by Ito and colleagues. They reported reduced activity for CYP1A2*4 (I386F), CYP1A2*6 (R431W), CYP1A2*8 (R456H), CYP1A2*11 (F186L), CYP1A2*15 (P42R), CYP1A2*16 (R377Q) and CYP1A2*21 (S298R and Y495Ter) toward phenacetin and 7-ethoxyresorufin substrates. The nonsense substitution (Y459Ter) of CYP1A2*21 results in a truncated protein that reduces the activity of the enzyme^25^. Moreover, two allelic variants CYP1A2*14 (T438I) and CYP1A2*20 (D436N) showed higher activity for phenacetin compared with wild-type enzyme. In the current study, P42R, R377Q, I386F, R431W and R456H variants were predicted as functional variants, of which R431W and R456H variants were also found to be associated with pathogenicity and cancer susceptibility.

Among nsSNPs predicted as cancer drivers, G73W and F432S were still voted by all methods even after applying more stringent thresholds. Accordingly, these variants were subjected to 200 ns MD simulations to explore the effect of substitutions on the protein structure. Findings demonstrated that these substitutions change protein structural features not only in proximity of the substituted residues but also in spatially distant regions. Both variants experienced a reduction in the number and strength of intramolecular hydrogen bonds as well as in β-sheet and α-helical content. Results derived from the principal component analysis (PCA) confirmed an increase in the overall flexibility especially for F432S variant. A drastic increase was also found for the CD loop flexibility which is a long serine-rich stretch (residues 148–158) extended into the solvent. Increased mobility of the CD loop has been recently reported upon simulation of R377Q^27^. In this regard, the experimentally observed loss of the enzymatic activity in R377Q variant has been attributed to the reduced heme stability due to the increased flexibility of the C helix, which is adjacent to the CD loop. The C helix is also adjacent to the heme prosthetic group and interacts with heme propionate oxygen via a conserved arginine residue (Arg137). Hence, any change in flexibility of the C helix could affect the stability of the heme. Moreover, C helix is one of the main regions involved in interaction with redox partners like cytochrome b5 (CYB5). CYPs binding to CYB5 is mediated through a groove on the proximal surface of the protein which includes C helix. There are also growing evidences for the involvement of CD loop in binding of some CYPs to the CYB5, although it appears the CYB5 interactive elements of various CYPs are type specific. For example, the interacting region on CYP3A4 in apo form consists of helices B, C, D, BB' and CD loops, β-bulge and meander region while on CYP2E1 is provided by helices C, J', L, β-bulge and meander region^63,64^. Taken together, it seems reasonable to expect that the high mobility of the CD loop in F432S may affect heme stability as well as interaction with CYB5.

## Conclusion

CYP1A2 is one of the main hepatic CYPs involved in the bioactivation of carcinogens and metabolism of clinically used drugs. Hence, nsSNPs of this enzyme could affect cancer susceptibility and drug efficiency. In current study, using a variety of computational tools, 38 out of 176 nsSNPs of human *CYP1A2* gene were predicted as functional variants. The functional nsSNPs were further analyzed to trace possible association with pathogenicity and cancer susceptibility. As a result, 18 nsSNPs predicted as pathogenic, of which G73R, G73W, R108Q, R108W, E168K, E346K, R431W, F432S and R456H variants were also found to be associated with hepatocellular carcinoma. We also performed 200 ns MD simulations to explore how G73W and F432S cancer driver variants affect the protein structure. Simulation results revealed several significant structural alterations, particularly for F432S variant. Among them, increased flexibility of the CD loop and loss of the hydrogen bond between heme and Arg137 from C helix were the most prominent ones, because they could affect the heme stability as well as the protein interaction with cytochrome b5. These findings may be considered in designing experimental studies and provide novel insights into understanding the structure–function relationship in CYP1A2 and other CYPs.

## Supplementary Information

Supplementary Information.
