# Supplementary material for: In silico screening and analysis of nonsynonymous SNPs in human CYP1A2 to assess possible associations with pathogenicity and cancer susceptibility
Source: Sci Rep. 2021 Mar 2;11:4977. doi: 10.1038/s41598-021-83696-x (PMC7925555; doi:10.1038/s41598-021-83696-x)
Supplement: Supplementary file 1 — Supplementary Information. [file 41598_2021_83696_MOESM1_ESM.doc]

***In silico* screening and analysis of nonsynonymous SNPs in human *CYP1A2* to assess possible associations with pathogenicity and cancer susceptibility**

Leila Navapour, Navid Mogharrab^*^

*Biophysics and Computational Biology Laboratory (BCBL), Department of Biology, College of Sciences, Shiraz University, Shiraz, Iran.*

**Corresponding author. Tel: +98 71 32273929, Fax: +98 713 228 0926*

*E-mail address:* [*mogharrab@shirazu.ac.ir*](mailto:mogharrab@shirazu.ac.ir)

**Contents**

**Supplementary Tables**

Table S1. The nsSNPs of human *CYP1A2* gene.

Table S2. Identification of functional nsSNPs of human CYP1A2.

Table S3. Hydrogen bonding network of the CD loop during last 120 ns.

**Supplementary Tables**

**Table S1. The nsSNPs of human *CYP1A2* gene.**

| **SNP identifier** | **Allele^1^ (R>V)^2^** | **AA (R>V)** | **Region** | **Validation status** |
| --- | --- | --- | --- | --- |
| rs765173274 | 75042081T>C | M1T | Exon 2 | frequency |
| rs201871401 | 75042087T>C | L3S | Exon 2 | cluster, frequency |
| rs372412769 | 75042108C>T | S10L | Exon 2 | cluster, frequency |
| rs60086777 | 75042122C>T | L15F | Exon 2 | 1000G, cluster, frequency |
| rs17861152 | 75042132C>G | S18C | Exon 2 | 1000G, cluster, frequency |
| rs17861152 | 75042132C>A | S18Y | Exon 2 | 1000G, cluster, frequency |
| rs1047643383 | 75042134G>C | A19P | Exon 2 | frequency |
| rs56160784 | 75042142C>G | F21L | Exon 2 | cluster, frequency |
| rs142454118 | 75042153T>G | F25C | Exon 2 | cluster, frequency |
| rs142454118 | 75042153T>C | F25S | Exon 2 | cluster, frequency |
| rs759309532 | 75042158G>A | V27M | Exon 2 | cluster, frequency |
| rs765154807 | 75042167G>T | G30C | Exon 2 | frequency |
| rs201934979 | 75042179C>T | R34W | Exon 2 | 1000G, cluster, frequency |
| rs146974121 | 75042185C>T | P36S | Exon 2 | cluster, frequency |
| rs146974121 | 75042185C>A | P36T | Exon 2 | cluster, frequency |
| rs751602658 | 75042189A>G | K37R | Exon 2 | frequency |
| rs200789139 | 75042198A>G | K40R | Exon 2 | cluster, frequency |
| rs72547511 | 75042204C>G | P42R | Exon 2 | no info |
| rs3743482 | 75042209G>A | E44K | Exon 2 | cluster, frequency, hapmap |
| rs201763966 | 75042221T>G | W48G | Exon 2 | 1000G, cluster, frequency |
| rs376605220 | 75042233G>A | G52R | Exon 2 | cluster |
| rs761818825 | 75042261C>T | P61L | Exon 2 | cluster, frequency |
| rs71651689 | 75042273T>C | L65P | Exon 2 | cluster, frequency |
| rs760996321 | 75042278A>G | R67G | Exon 2 | frequency |
| rs755565165 | 75042290C>T | R71C | Exon 2 | cluster, frequency |
| rs779505412 | 75042291G>A | R71H | Exon 2 | frequency |
| rs45565238 | 75042296G>A | G73R | Exon 2 | 1000G, cluster, frequency |
| rs45565238 | 75042296G>T | G73W | Exon 2 | 1000G, cluster, frequency |
| rs150164960 | 75042302G>A | V75I | Exon 2 | 1000G, cluster, frequency |
| rs761941661 | 75042314C>T | R79C | Exon 2 | frequency |
| rs752037611 | 75042315G>A | R79H | Exon 2 | frequency |
| rs752037611 | 75042315G>T | R79L | Exon 2 | frequency |
| rs138652540 | 75042327C>T | T83M | Exon 2 | 1000G, cluster, frequency |
| rs759912942 | 75042332G>C | V85L | Exon 2 | frequency |
| rs759912942 | 75042332G>A | V85M | Exon 2 | frequency |
| rs556218799 | 75042347C>T | R90C | Exon 2 | 1000G, frequency |
| rs201551575 | 75042348G>A | R90H | Exon 2 | 1000G, cluster, frequency |
| rs201551575 | 75042348G>T | R90L | Exon 2 | 1000G, cluster, frequency |
| rs779502284 | 75042357C>T | T93I | Exon 2 | frequency |
| rs754758658 | 75042363G>T | R95L | Exon 2 | frequency |
| rs754758658 | 75042363G>A | R95Q | Exon 2 | frequency |
| rs749152552 | 75042362C>T | R95W | Exon 2 | frequency |
| rs545726003 | 75042368G>A | A97T | Exon 2 | 1000G |
| rs773366123 | 75042372T>A | L98Q | Exon 2 | frequency |
| rs201650099 | 75042378G>A | R100Q | Exon 2 | cluster, frequency |
| rs145266863 | 75042377C>T | R100W | Exon 2 | cluster, frequency |
| rs763535401 | 75042386G>A | D103N | Exon 2 | frequency |
| rs34067076 | 75042389G>A | D104N | Exon 2 | 1000G, cluster, frequency |
| rs376179316 | 75042402G>A | R108Q | Exon 2 | cluster, frequency |
| rs766082944 | 75042401C>T | R108W | Exon 2 | frequency |
| rs45442197 | 75042410C>T | L111F | Exon 2 | 1000G, cluster, frequency |
| rs201820772 | 75042419T>C | S114P | Exon 2 | cluster, frequency |
| rs377141209 | 75042425C>T | L116F | Exon 2 | cluster, frequency |
| rs763157275 | 75042450C>T | T124I | Exon 2 | frequency |
| rs141543251 | 75042454C>A | F125L | Exon 2 | 1000G, cluster, frequency |
| rs760284408 | 75042471C>T | P131L | Exon 2 | cluster, frequency |
| rs368187861 | 75042485C>T | R136C | Exon 2 | cluster, frequency |
| rs150893756 | 75042486G>A | R136H | Exon 2 | cluster, frequency |
| rs139412032 | 75042489G>A | R137Q | Exon 2 | cluster, |
| rs757176570 | 75042491C>T | R138C | Exon 2 | frequency |
| rs59410695 | 75042492G>A | R138H | Exon 2 | cluster, frequency |
| rs192799115 | 75042527G>A | A150T | Exon 2 | 1000G, cluster, frequency |
| rs763825636 | 75042539G>A | A154T | Exon 2 | frequency |
| rs781205661 | 75042573T>G | V165G | Exon 2 | frequency |
| rs72547512 | 75042581G>A | E168K | Exon 2 | cluster |
| rs72547512 | 75042581G>C | E168Q | Exon 2 | cluster |
| rs756244484 | 75042591C>A | A171D | Exon 2 | frequency |
| rs56075956 | 75042617A>G | M180V | Exon 2 | cluster, frequency |
| rs147248980 | 75042634C>G | H185Q | Exon 2 | cluster, frequency |
| rs72547513 | 75042637C>A | F186L | Exon 2 | cluster, frequency |
| rs369511887 | 75042638G>A | D187N | Exon 2 | 1000G, cluster, frequency |
| rs140747247 | 75042651A>C | Q191P | Exon 2 | 1000G, cluster, frequency |
| rs144697774 | 75042674G>A | V199I | Exon 2 | cluster, frequency |
| rs750360601 | 75042680G>C | G201R | Exon 2 | frequency |
| rs750360601 | 75042680G>A | G201S | Exon 2 | frequency |
| rs45540640 | 75042692T>G | F205V | Exon 2 | 1000G, cluster, frequency |
| rs200836818 | 75042695G>A | G206R | Exon 2 | cluster, frequency |
| rs202191520 | 75042708C>T | P210L | Exon 2 | 1000G, cluster, frequency |
| rs758748797 | 75042713A>T | S212C | Exon 2 | no info |
| rs747544962 | 75042719G>A | D214N | Exon 2 | cluster |
| rs761744781 | 75042746A>G | T223A | Exon 2 | frequency |
| rs760714274 | 75042754G>C | E225D | Exon 2 | frequency |
| rs373580935 | 75042758G>A | V227M | Exon 2 | cluster, frequency |
| rs765435682 | 75042767G>C | A230P | Exon 2 | frequency |
| rs201537008 | 75042776G>A | G233R | Exon 2 | 1000G, cluster, frequency |
| rs200328907 | 75042782C>G | P235A | Exon 2 | 1000G, cluster, frequency |
| rs200328907 | 75042782C>T | P235S | Exon 2 | 1000G, cluster, frequency |
| rs200571120 | 75042792T>C | F238S | Exon 2 | cluster, frequency |
| rs375045680 | 75042806C>T | R243C | Exon 2 | cluster, frequency |
| rs542879792 | 75042820C>G | N247K | Exon 2 | 1000G, frequency |
| rs575035489 | 75042825C>T | A249V | Exon 2 | cluster, frequency |
| rs561167723 | 75042848A>T | N257Y | Exon 2 | 1000G |
| rs370942811 | 75042905G>C | D276H | Exon 2 | cluster, frequency |
| rs370942811 | 75042905G>A | D276N | Exon 2 | cluster, frequency |
| rs267604321 | 75043540G>A | R281Q | Exon 3 | cluster, frequency |
| rs45468096 | 75043539C>T | R281W | Exon 3 | 1000G, cluster, frequency |
| rs368776150 | 75043549C>T | T284M | Exon 3 | cluster, frequency |
| rs745581564 | 75043554G>T | A286S | Exon 3 | frequency |
| rs745581564 | 75043554G>A | A286T | Exon 3 | frequency |
| rs774252909 | 75043564A>G | K289R | Exon 3 | frequency |
| rs771876603 | 75043567A>G | H290R | Exon 3 | cluster, frequency |
| rs17861157 | 75043592C>A | S298R | Exon 3 | 1000G, cluster, frequency |
| rs35796837 | 75043593G>A | G299S | Exon 3 | 1000G, cluster, frequency |
| rs28399418 | 75043638A>G | I314V | Exon 3 | cluster, frequency |
| rs778097570 | 75044124C>T | T324I | Exon 4 | frequency |
| rs778097570 | 75044124C>G | T324R | Exon 4 | frequency |
| rs200303014 | 75044145T>G | M331R | Exon 4 | cluster, frequency |
| rs200303014 | 75044145T>C | M331T | Exon 4 | cluster, frequency |
| rs369659890 | 75044151T>C | L333P | Exon 4 | cluster, frequency |
| rs765952249 | 75044173G>T | Q340H | Exon 4 | frequency |
| rs574824024 | 75044189G>A | E346K | Exon 4 | 1000G, frequency |
| rs56276455 | 75044195G>A | D348N | Exon 4 | cluster, frequency |
| rs764121366 | 75044471T>G | V350G | Exon 5 | frequency |
| rs144076129 | 75044486G>A | R355Q | Exon 5 | cluster, frequency |
| rs148157092 | 75044485C>T | R355W | Exon 5 | 1000G, cluster, frequency |
| rs55918015 | 75044489G>A | R356Q | Exon 5 | 1000G, cluster, frequency |
| rs147333000 | 75044488C>T | R356W | Exon 5 | 1000G, cluster, frequency |
| rs756307932 | 75044491C>G | P357A | Exon 5 | frequency |
| rs769113145 | 75044495G>A | R358Q | Exon 5 | frequency |
| rs749598098 | 75044494C>T | R358W | Exon 5 | frequency |
| rs574726215 | 75044505C>G | D361E | Exon 5 | 1000G |
| rs776078971 | 75044518C>T | P366S | Exon 5 | frequency |
| rs72547515 | 75044552G>A | R377Q | Exon 5 | no info |
| rs751510035 | 75044558C>A | S379Y | Exon 5 | frequency |
| rs767723757 | 75044567T>G | L382W | Exon 5 | frequency |
| rs750455376 | 75044576C>A | T385N | Exon 5 | frequency |
| rs72547516 | 75044578A>T | I386F | Exon 5 | 1000G, cluster, frequency |
| rs72547516 | 75044578A>G | I386V | Exon 5 | 1000G, cluster, frequency |
| rs557412265 | 75044584C>T | H388Y | Exon 5 | 1000G, frequency |
| rs774289191 | 75045526A>G | T390A | Exon 6 | frequency |
| rs911014711 | 75045541A>G | T395A | Exon 6 | frequency |
| rs149928755 | 75045542C>T | T395M | Exon 6 | 1000G, cluster, frequency |
|  | 75045547A>C | N397H | Exon 6 | no info |
| rs866111643 | 75045562C>T | P402S | Exon 6 | cluster |
| rs55889066 | 75045575G>A | C406Y | Exon 6 | cluster |
| rs759768496 | 75045578T>C | V407A | Exon 6 | frequency |
| rs759768496 | 75045578T>A | V407D | Exon 6 | frequency |
| rs752138293 | 75045584T>A | V409E | Exon 6 | frequency |
| rs377527644 | 75045583G>A | V409I | Exon 6 | cluster, frequency |
| rs748737498 | 75045599T>G | V414G | Exon 6 | frequency |
| rs376674911 | 75045598G>A | V414I | Exon 6 | cluster, frequency |
| rs376674911 | 75045598G>C | V414L | Exon 6 | cluster, frequency |
| rs768486827 | 75045611C>T | P418L | Exon 6 | frequency |
| rs374433914 | 75047156G>C | E426D | Exon 7 | cluster |
| rs145557631 | 75047161G>T | R428L | Exon 7 | 1000G, cluster, frequency |
| rs145557631 | 75047161G>A | R428Q | Exon 7 | 1000G, cluster, frequency |
| rs149710194 | 75047160C>T | R428W | Exon 7 | cluster, frequency |
| rs28399424 | 75047169C>T | R431W | Exon 7 | 1000G, cluster, frequency |
| rs201485133 | 75047173T>C | F432S | Exon 7 | 1000G, cluster |
| rs770969636 | 75047178A>C | T434P | Exon 7 | cluster, frequency |
| rs775498266 | 75047185A>G | D436G | Exon 7 | frequency |
| rs144148965 | 75047184G>A | D436N | Exon 7 | 1000G, cluster, frequency |
| rs45486893 | 75047191C>T | T438I | Exon 7 | 1000G, cluster, frequency |
| rs45486893 | 75047191C>A | T438N | Exon 7 | 1000G, cluster, frequency |
| rs140757511 | 75047210G>T | L444F | Exon 7 | cluster, frequency |
| rs566851431 | 75047218A>T | K447M | Exon 7 | 1000G, frequency |
| rs367858322 | 75047244C>T | R456C | Exon 7 | 1000G, cluster, frequency |
| rs72547517 | 75047245G>A | R456H | Exon 7 | cluster, frequency |
| rs745824533 | 75047248G>C | R457P | Exon 7 | frequency |
| rs745824533 | 75047248G>A | R457Q | Exon 7 | frequency |
| rs34151816 | 75047247C>T | R457W | Exon 7 | 1000G, cluster |
| rs772597894 | 75047259G>A | E461K | Exon 7 | frequency |
| rs199528490 | 75047262G>C | V462L | Exon 7 | 1000G, cluster, frequency |
| rs780975619 | 75047296C>A | A473D | Exon 7 | frequency |
| rs143028942 | 75047325G>A | V483M | Exon 7 | cluster, frequency |
| rs773716931 | 75047332C>T | P485L | Exon 7 | cluster |
| rs140211191 | 75047335G>A | G486D | Exon 7 | cluster, frequency |
| rs571663822 | 75047337G>A | V487M | Exon 7 | 1000G, cluster, frequency |
| rs755880133 | 75047364G>A | G496R | Exon 7 | frequency |
| rs780006160 | 75047371C>A | T498N | Exon 7 | frequency |
| rs754897375 | 75047386G>A | R503H | Exon 7 | frequency |
| rs778132807 | 75047404C>A | A509E | Exon 7 | frequency |
| rs778132807 | 75047404C>T | A509V | Exon 7 | frequency |
| rs374094758 | 75047407G>T | R510L | Exon 7 | cluster |
| rs374094758 | 75047407G>A | R510Q | Exon 7 | cluster |
| rs138459442 | 75047406C>T | R510W | Exon 7 | cluster, frequency |

^1^ NC_000015.9, GRCh37.p13 (Annotation release 105)

^2^ Reference>Variant

**Table S2.** Identification of functional nsSNPs of human CYP1A2.

| **SNP** | **SIFT** | **PROVEAN** | **M. Assessor** | **FATHMM-MKL** | **LRT** | **EFIN** | **CADD** | **PolyPhen2** | **SNAP2** |
| --- | --- | --- | --- | --- | --- | --- | --- | --- | --- |
| M1T | F | N | ― | F | N | N | F | F | N |
| L3S | F | N | F | N | N | N | N | F | N |
| S10L | F | N | F | F | N | N | F | N | N |
| L15F | F | N | F | F | F | N | F | F | N |
| S18C | N | N | F | N | N | N | F | F | F |
| S18Y | F | N | F | N | N | F | N | F | N |
| A19P | N | N | F | N | N | N | N | F | N |
| F21L | N | N | F | F | F | N | F | F | N |
| F25C | F | F | F | F | N | N | F | F | N |
| F25S | F | F | F | F | F | N | F | F | N |
| V27M | F | N | F | N | N | N | N | F | N |
| G30C | N | N | N | N | N | N | N | F | N |
| R34W | N | F | F | N | N | N | N | N | N |
| **P36S** | **F** | **F** | **F** | **F** | **F** | **F** | **F** | **F** | **F** |
| P36T | F | F | F | F | F | F | F | F | N |
| K37R | N | N | F | F | N | N | N | N | N |
| K40R | N | N | N | F | F | N | N | F | N |
| **P42R** | **F** | **F** | **F** | **F** | **F** | **F** | **F** | **F** | **F** |
| E44K | F | N | N | F | F | N | F | N | F |
| W48G | F | F | F | F | N | F | F | F | F |
| **G52R** | **F** | **F** | **F** | **F** | **F** | **F** | **F** | **F** | **F** |
| **P61L** | **F** | **F** | **F** | **F** | **F** | **F** | **F** | **F** | **F** |
| **L65P** | **F** | **F** | **F** | **F** | **F** | **F** | **F** | **F** | **F** |
| R67G | N | F | F | N | N | N | N | N | N |
| R71C | F | F | F | N | N | N | F | N | N |
| R71H | F | N | F | N | N | N | N | N | N |
| **G73R** | **F** | **F** | **F** | **F** | **F** | **F** | **F** | **F** | **F** |
| **G73W** | **F** | **F** | **F** | **F** | **F** | **F** | **F** | **F** | **F** |
| V75I | N | N | F | F | F | N | F | N | N |
| **R79C** | **F** | **F** | **F** | **F** | **F** | **F** | **F** | **F** | **F** |
| R79H | N | N | N | F | F | N | F | N | N |
| R79L | F | F | F | F | F | N | F | F | F |
| T83M | N | N | F | N | F | N | F | F | N |
| V85L | F | N | F | F | F | F | F | F | F |
| **V85M** | **F** | **F** | **F** | **F** | **F** | **F** | **F** | **F** | **F** |
| R90C | F | N | N | F | F | N | F | F | F |
| R90H | F | N | N | F | F | N | F | F | F |
| R90L | F | N | N | F | F | N | F | N | F |
| T93I | F | F | F | F | F | F | F | F | N |
| R95L | F | F | F | N | F | F | F | F | F |
| R95Q | F | N | F | N | F | N | F | N | N |
| R95W | F | F | F | N | N | F | F | F | F |
| A97T | F | F | F | F | F | F | F | F | N |
| **L98Q** | **F** | **F** | **F** | **F** | **F** | **F** | **F** | **F** | **F** |
| R100Q | N | N | N | N | N | N | F | N | N |
| R100W | F | F | F | F | N | F | F | F | F |
| D103N | F | F | F | F | N | N | N | F | N |
| D104N | N | F | F | F | F | N | F | F | N |
| **R108Q** | **F** | **F** | **F** | **F** | **F** | **F** | **F** | **F** | **F** |
| **R108W** | **F** | **F** | **F** | **F** | **F** | **F** | **F** | **F** | **F** |
| L111F | N | F | N | F | N | N | N | F | F |
| S114P | F | N | N | F | N | N | F | F | F |
| L116F | N | N | N | N | N | N | N | N | N |
| T124I | F | F | N | F | N | N | N | N | F |
| **F125L** | **F** | **F** | **F** | **F** | **F** | **F** | **F** | **F** | **F** |
| P131L | N | F | F | F | F | N | F | F | N |
| **R136C** | **F** | **F** | **F** | **F** | **F** | **F** | **F** | **F** | **F** |
| R136H | N | N | N | F | F | N | F | F | N |
| **R137Q** | **F** | **F** | **F** | **F** | **F** | **F** | **F** | **F** | **F** |
| **R138C** | **F** | **F** | **F** | **F** | **F** | **F** | **F** | **F** | **F** |
| R138H | F | F | F | F | N | N | F | F | F |
| A150T | N | N | F | F | F | N | F | F | N |
| A154T | N | N | N | N | N | N | N | N | N |
| **V165G** | **F** | **F** | **F** | **F** | **F** | **F** | **F** | **F** | **F** |
| **E168K** | **F** | **F** | **F** | **F** | **F** | **F** | **F** | **F** | **F** |
| E168Q | F | N | F | F | F | F | F | F | F |
| A171D | N | N | N | N | N | N | N | N | N |
| M180V | F | N | F | F | F | N | N | F | N |
| H185Q | N | N | F | N | N | N | N | N | N |
| F186L | F | F | F | N | F | F | F | F | F |
| D187N | N | F | N | F | F | N | N | N | N |
| Q191P | N | N | N | N | N | N | N | F | N |
| V199I | N | N | N | F | F | N | N | N | N |
| G201R | F | N | F | F | F | F | F | F | F |
| G201S | N | N | N | F | F | N | N | N | N |
| **F205V** | **F** | **F** | **F** | **F** | **F** | **F** | **F** | **F** | **F** |
| G206R | F | F | F | F | N | F | F | F | F |
| P210L | F | N | F | N | N | N | F | F | N |
| S212C | F | F | N | N | N | N | N | F | N |
| D214N | N | N | N | N | N | N | N | N | N |
| T223A | N | N | N | N | F | N | N | N | N |
| E225D | N | N | N | F | N | N | N | N | N |
| V227M | N | N | F | N | F | N | F | F | N |
| A230P | F | N | F | F | F | N | F | F | F |
| G233R | F | F | F | F | F | N | F | F | F |
| P235A | N | F | N | F | N | N | N | N | N |
| P235S | F | F | F | F | F | F | F | F | N |
| **F238S** | **F** | **F** | **F** | **F** | **F** | **F** | **F** | **F** | **F** |
| **R243C** | **F** | **F** | **F** | **F** | **F** | **F** | **F** | **F** | **F** |
| N247K | F | F | F | N | F | N | F | F | F |
| A249V | N | N | N | N | N | N | N | F | N |
| N257Y | N | F | F | F | F | F | F | F | N |
| D276H | F | F | F | F | F | F | F | F | N |
| D276N | N | F | N | F | N | N | F | N | N |
| R281Q | N | F | N | N | F | N | F | N | F |
| R281W | F | F | F | F | N | F | F | F | F |
| T284M | F | F | F | F | N | N | F | F | N |
| A286S | N | N | N | F | F | N | N | N | N |
| A286T | F | N | F | F | F | N | F | F | N |
| K289R | N | N | N | F | N | N | N | N | N |
| H290R | N | F | N | F | F | N | N | N | N |
| S298R | N | N | F | N | N | N | N | N | N |
| G299S | N | N | N | N | N | N | N | N | N |
| I314V | F | N | F | F | F | N | N | F | N |
| **T324I** | **F** | **F** | **F** | **F** | **F** | **F** | **F** | **F** | **F** |
| **T324R** | **F** | **F** | **F** | **F** | **F** | **F** | **F** | **F** | **F** |
| M331R | F | F | F | F | N | F | F | F | N |
| M331T | F | F | N | F | N | N | F | N | N |
| L333P | F | F | F | F | N | F | F | F | F |
| Q340H | F | F | F | F | N | F | F | F | N |
| **E346K** | **F** | **F** | **F** | **F** | **F** | **F** | **F** | **F** | **F** |
| D348N | F | F | F | F | N | N | F | N | N |
| V350G | N | N | F | N | N | N | N | F | N |
| **R355Q** | **F** | **F** | **F** | **F** | **F** | **F** | **F** | **F** | **F** |
| **R355W** | **F** | **F** | **F** | **F** | **F** | **F** | **F** | **F** | **F** |
| R356Q | N | N | N | N | N | N | N | N | N |
| R356W | N | N | F | N | N | N | N | N | N |
| P357A | F | F | F | F | F | N | F | F | F |
| R358Q | N | N | N | N | N | N | F | N | N |
| R358W | F | F | F | N | N | F | F | N | F |
| D361E | F | F | F | F | N | F | F | N | F |
| P366S | F | F | F | F | F | F | F | F | N |
| **R377Q** | **F** | **F** | **F** | **F** | **F** | **F** | **F** | **F** | **F** |
| S379Y | F | F | F | F | F | F | F | F | N |
| L382W | F | F | F | F | N | N | F | F | F |
| T385N | F | F | N | F | F | F | F | F | N |
| **I386F** | **F** | **F** | **F** | **F** | **F** | **F** | **F** | **F** | **F** |
| I386V | F | N | N | F | N | N | N | N | N |
| **H388Y** | **F** | **F** | **F** | **F** | **F** | **F** | **F** | **F** | **F** |
| T390A | F | F | N | F | F | N | F | F | N |
| T395A | N | N | F | N | N | N | N | N | N |
| T395M | F | N | F | N | N | N | F | F | N |
| N397H | F | F | F | F | F | N | F | F | N |
| P402S | F | F | F | F | N | F | F | F | N |
| C406Y | F | F | F | F | N | N | N | N | F |
| V407A | F | F | F | F | N | N | N | F | F |
| V407D | F | F | F | F | N | N | F | F | F |
| V409E | F | F | F | F | N | N | F | F | F |
| V409I | N | N | N | N | N | N | N | N | N |
| V414G | F | F | F | F | N | N | F | F | F |
| V414I | N | N | N | F | N | N | N | N | N |
| V414L | N | N | F | F | N | N | F | N | F |
| P418L | F | F | F | F | N | N | N | F | N |
| E426D | N | N | N | N | N | N | N | N | N |
| R428L | N | F | F | F | N | N | N | N | N |
| R428Q | N | N | F | N | N | N | N | F | N |
| R428W | N | F | F | F | N | N | F | F | F |
| **R431W** | **F** | **F** | **F** | **F** | **F** | **F** | **F** | **F** | **F** |
| **F432S** | **F** | **F** | **F** | **F** | **F** | **F** | **F** | **F** | **F** |
| T434P | F | N | F | F | F | F | N | F | N |
| D436G | F | F | F | N | N | N | N | N | F |
| D436N | N | N | N | N | N | N | N | N | N |
| T438I | F | F | F | N | N | N | N | F | F |
| T438N | F | N | N | N | N | N | N | N | F |
| L444F | F | N | F | N | N | N | F | F | F |
| **K447M** | **F** | **F** | **F** | **F** | **F** | **F** | **F** | **F** | **F** |
| R456C | F | F | F | F | N | F | F | F | F |
| **R456H** | **F** | **F** | **F** | **F** | **F** | **F** | **F** | **F** | **F** |
| **R457P** | **F** | **F** | **F** | **F** | **F** | **F** | **F** | **F** | **F** |
| R457Q | F | N | F | F | F | N | F | F | F |
| **R457W** | **F** | **F** | **F** | **F** | **F** | **F** | **F** | **F** | **F** |
| **E461K** | **F** | **F** | **F** | **F** | **F** | **F** | **F** | **F** | **F** |
| V462L | N | N | F | N | N | N | N | N | N |
| **A473D** | **F** | **F** | **F** | **F** | **F** | **F** | **F** | **F** | **F** |
| V483M | N | N | F | N | N | N | N | F | N |
| P485L | F | F | F | N | N | N | F | N | N |
| G486D | F | F | F | F | F | N | N | F | N |
| V487M | N | N | F | N | N | N | N | F | N |
| G496R | F | F | F | F | N | F | F | F | F |
| **T498N** | **F** | **F** | **F** | **F** | **F** | **F** | **F** | **F** | **F** |
| R503H | N | N | N | N | N | N | N | N | N |
| A509E | F | N | F | F | N | N | F | F | F |
| A509V | N | N | N | N | N | N | N | N | N |
| R510L | F | N | N | N | N | N | N | N | F |
| R510Q | N | N | N | N | N | N | N | N | N |
| R510W | N | N | N | N | N | N | N | N | F |

F: Functional, N: Non-functional/Neutral.

**Table S3.** Hydrogen bonding network of the CD loop during last 120 ns.

|  |  | **Occupancy (%)** | | |
| --- | --- | --- | --- | --- |
| **Donor** | **Acceptor** | **WT** | **G73W** | **F432S** |
| ASP152 (N) | SER158 (O) | 96.62 | 96.44 | ― |
| ALA154 (N) | ASP152 (OD1) | 55.54 | 93.57 | ― |
| ALA154 (N) | ASP152 (OD2) | 42.53 | 4.34 | ― |
| SER155 (N) | ASP152 (O) | 76.23 | 71.19 | 6.86 |
| Ser155 (OG) | Glu163 (OE1) | 41.04 | 31.05 | ― |
| Ser155 (OG) | Glu163 (OE2) | 41.97 | 39.62 | ― |
| Cys159 (N) | Glu162 (OE1) | 29.42 | 39.87 | 1.67 |
| Cys159 (N) | Glu162 (OE2) | 61.88 | 47.23 | 0.21 |
| Leu161 (N) | Ser148 (O) | 97.75 | 97.56 | ― |
| Arg281 (NH1) | Asp152 (OD1) | 55.82 | 92.90 | 2.86 |
| Arg281 (NH1) | Asp152 (OD2) | 47.15 | 12.00 | 6.67 |
| Arg281 (NH2) | Asp152 (OD1) | 64.03 | 45.91 | 5.94 |
| Arg281 (NH2) | Asp152 (OD2) | 73.34 | 92.74 | 6.95 |
